# Understanding inter-individual variability of experimental pain habituation and conditioned pain modulation in healthy individuals

**DOI:** 10.1038/s41598-024-73158-5

**Published:** 2024-09-27

**Authors:** Iara De Schoenmacker, Paulina S. Scheuren, Laura Sirucek, Robin Lütolf, Lindsay M. Gorrell, Jan Rosner, Armin Curt, Petra Schweinhardt, Michèle Hubli

**Affiliations:** 1 Spinal Cord Injury Center, Balgrist University Hospital, University of Zurich, Forchstrasse 340, 8008 Zurich, Switzerland; 2 Biomedical Data Science Lab, Institute of Translational Medicine, Swiss Federal Institute of Technology (ETH) Zurich, Zurich, Switzerland; 3 Department of Neurology, University Hospital Bern, Inselspital, University of Bern, Bern, Switzerland; 4 International Collaboration on Repair Discoveries, University of British Columbia, Vancouver, BC Canada; 5 Department of Anesthesiology, Pharmacology and Therapeutics, Faculty of Medicine, University of British Columbia, Vancouver, BC Canada; 6 Department of Chiropractic Medicine, Integrative Spinal Research Group, Balgrist University Hospital, University of Zurich, Zurich, Switzerland; 7 Neuroscience Center Zurich (ZNZ), Zurich, Switzerland; 8 Danish Pain Research Center, Department of Clinical Medicine, Aarhus University, Aarhus, Denmark

**Keywords:** Sensory processing, Somatosensory system, Neurophysiology, Diagnostic markers

## Abstract

Although reduced experimental pain habituation is proposed as a proxy of diminished endogenous pain modulatory capacity in chronic pain, prior studies show contradictory findings. Even across healthy participants, pain habituation varies substantially, which may relate to another measure of endogenous pain modulation, i.e., conditioned pain modulation (CPM). Hence, this study investigated the relationship between pain habituation and CPM. Pain habituation was assessed in 45 healthy participants between two blocks of 15–20 contact-heat stimuli applied to the hand. Habituation of subjective pain ratings and objective neurophysiological readouts (contact-heat evoked potential (CHEP) and palmar sympathetic skin response (SSR)) was investigated. CPM was assessed by comparing heat pain thresholds before and after hand immersion in a noxious cold (9 °C) and lukewarm water bath (32 °C, to control for repeated measures effects). Pain habituation showed a large variability, with subjective but not objective pain habituation correlating with cold-induced CPM effects (r = 0.50; *p* = 0.025). This correlation was not observed for ‘true’ CPM effects (corrected for repeated measures effects) nor for CPM effects induced by a lukewarm water bath. These findings suggest that the observed variability in subjective pain habituation may be influenced by both descending endogenous pain modulation and peripheral adaptation processes associated with repeated measures. Objective pain habituation readouts, i.e., CHEPs and SSRs, capture different, complementary aspects of endogenous pain modulation.

## Introduction

Acute pain is an important warning signal that alerts us to potential tissue damage or injury. In contrast, chronic pain loses its protective function and can persist long after the original injury or tissue damage has healed^1^. Chronic pain is thought to be associated with sensitization along the nociceptive neuraxis^1^, including a pronociceptive state or decrement in endogenous pain modulation^2^. Experimental pain habituation, characterized by a decrease in response (e.g., pain ratings) to repeated noxious stimuli^3^, has repeatedly been reported to be reduced, especially in patients with different chronic headache disorders (for review see^4^). However, in many other chronic pain conditions, such as chronic low back pain, the literature to date shows mixed results, with many studies demonstrating no difference in pain habituation between individuals with chronic pain and healthy participants^4^. Even within healthy participants, a large inter-individual variability in pain habituation is observed^5^, making it difficult to convincingly associate reduced pain habituation with a pronociceptive state of endogenous pain modulation. The underlying physiological mechanism of habituation to noxious stimuli is still controversial^6^. Previous fMRI studies have suggested that pain habituation and its associated attenuation of blood oxygenation level dependent signals in brain areas such as the anterior cingulate cortex (ACC) may be related to descending pain inhibition^7,8^. A frequently used psychophysical assessment of descending pain modulatory capacities in humans is conditioned pain modulation (CPM).

Both pain habituation^5^ and CPM^9^ can be reliably measured in individual participants. Moreover, Treister and colleagues^10^ previously demonstrated that pain habituation in healthy participants positively correlated with CPM. However, this study only included subjective readouts of pain habituation. To gain a more comprehensive understanding of pain habituation, our study incorporated objective readouts of pain habituation including neurophysiological and autonomic readouts^5^. To date, such objective neurophysiological and autonomic habituation have often shown different habituation patterns than subjective pain ratings^5,11,12^. For example, our previous work showed more profound habituation of autonomic readouts (i.e., sympathetic skin response (SSR)) compared to pain ratings and neurophysiological readouts in healthy participants^5^, which may be attributed to the additional involvement of spinal and bulbar processes in sympathetic skin responses^13^. Other studies even reported contrasting response patterns between subjective and objective habituation readouts. On the one hand, participants showed sensitization based on subjective pain ratings after consecutive heat stimuli. On the other hand, they presented with habituation of objective pain-induced sympathetic skin responses^11^. Eitner and colleagues^14^ compared habituation and CPM effects on subjective and objective habituation readouts, namely pain ratings and brain evoked potentials, demonstrating that CPM effects extend beyond mere pain habituation. While their research examined the contribution of pain habituation to the CPM effect, our study explores how the variability in pain habituation can be explained by the individuals’ CPM effect.

Pain habituation may also depend on inter-individual differences in psychological traits such as anxiety, depression, and pain catastrophizing^15,16^. This could be attributed to the fact that brain structures involved in pain habituation, such as the insula and rostral anterior cingulate cortex^17^, are also involved in processing affective aspects of pain, stress and anxiety^18,19^. Therefore, we exploratively examined the relationship between pain habituation and signs of anxiety, depression and pain catastrophizing. Lastly, it has been previously reported that biological factors such as age and sex influence pain habituation^20,21^. Young and male participants showed stronger pain habituation than elderly^20^ and female participants^21^, respectively.

The primary aim of this study was to investigate the relationship between different pain habituation readouts, i.e., subjective pain ratings, neurophysiological and autonomic readouts, and the CPM capacity. In addition, we wanted to gain a more nuanced understanding of the biopsychological factors (i.e., age, sex, anxiety, depression, and pain catastrophizing) that potentially contribute to inter-individual variability in pain habituation of healthy individuals. We hypothesized that more pronounced pain habituation, both subjective and objective, correlates with stronger inhibitory CPM effects. Also, we hypothesized that this more pronounced pain habituation is associated with lower scores in questionnaires related to anxiety, depression, and pain catastrophizing. With regard to biological/demographic factors, we expected that younger and male participants would show stronger pain habituation than older and female participants, respectively.

## Methods

### Participants and study protocol

This manuscript includes a secondary analysis of a larger study funded by the Clinical Research Priority Program (CRPP) Pain from the University of Zurich comprising a comprehensive test battery (2 visits of 3 h each) including 101 chronic pain patients and 63 age- and sex-matched healthy controls. The larger study included the evaluation of clinical pain characteristics, neurophysiological assessments, experimental pain paradigms, and psychological and pain questionnaires. In line with the aim of investigating the physiological inter-individual variability of pain habituation, the data reported in this manuscript are based solely on the data from the healthy control cohort, with the hand as the testing area, following the same stimulation protocol (N = 45). The included data are: (1) psychological questionnaires (i.e., Pain Catastrophizing Scale (PCS)^22^ and Hospital Anxiety and Depression Scale (HADS)), (2) heat pain threshold (HPT) in the testing area assessed in accordance with the quantitative sensory testing (QST) protocol provided by the German Research Network on Neuropathic Pain (DFNS)^23^ to determine baseline heat pain sensitivity, (3) experimental pain habituation, and (4) CPM. The psychological questionnaires were completed electronically. The PCS is a compound score of 13 items, with total scores ranging from 0 to 52. A score above 30 is considered indicative of a clinically significant level of catastrophizing. The HADS consists of 14 items divided into two subscales: one for anxiety and one for depression, each containing 7 items. Each subscale has a scoring range from 0 to 21, where scores between 8 and 10 suggest a moderate likelihood, and scores from 11 to 21 suggest a high likelihood, of a mood disorder. Experimental pain habituation was randomly assessed in the beginning of the first (N = 11) or second visit (N = 34) and CPM in the end of the second visit. Exclusion criteria for all participants were acute/subacute pain or a history of chronic pain (> 3 months), neurological diseases (e.g., polyneuropathy), systemic diseases (e.g., diabetes or autoimmune disease), clinically manifested mental illnesses, the inability to adhere to research instructions, or pregnancy. Written informed consent was obtained from each participant and all experimental procedures were conducted in accordance with the Declaration of Helsinki. The study was approved by the local ethics board ‘Kantonale Ethikkommission Zürich, KEK’ (EK-04/2006, PB_2016-02,051 and PB_2019-00,136, clinicaltrial.gov number: NCT02138344 and NCT04433299).

### Experimental pain habituation

**Fig. 1:**
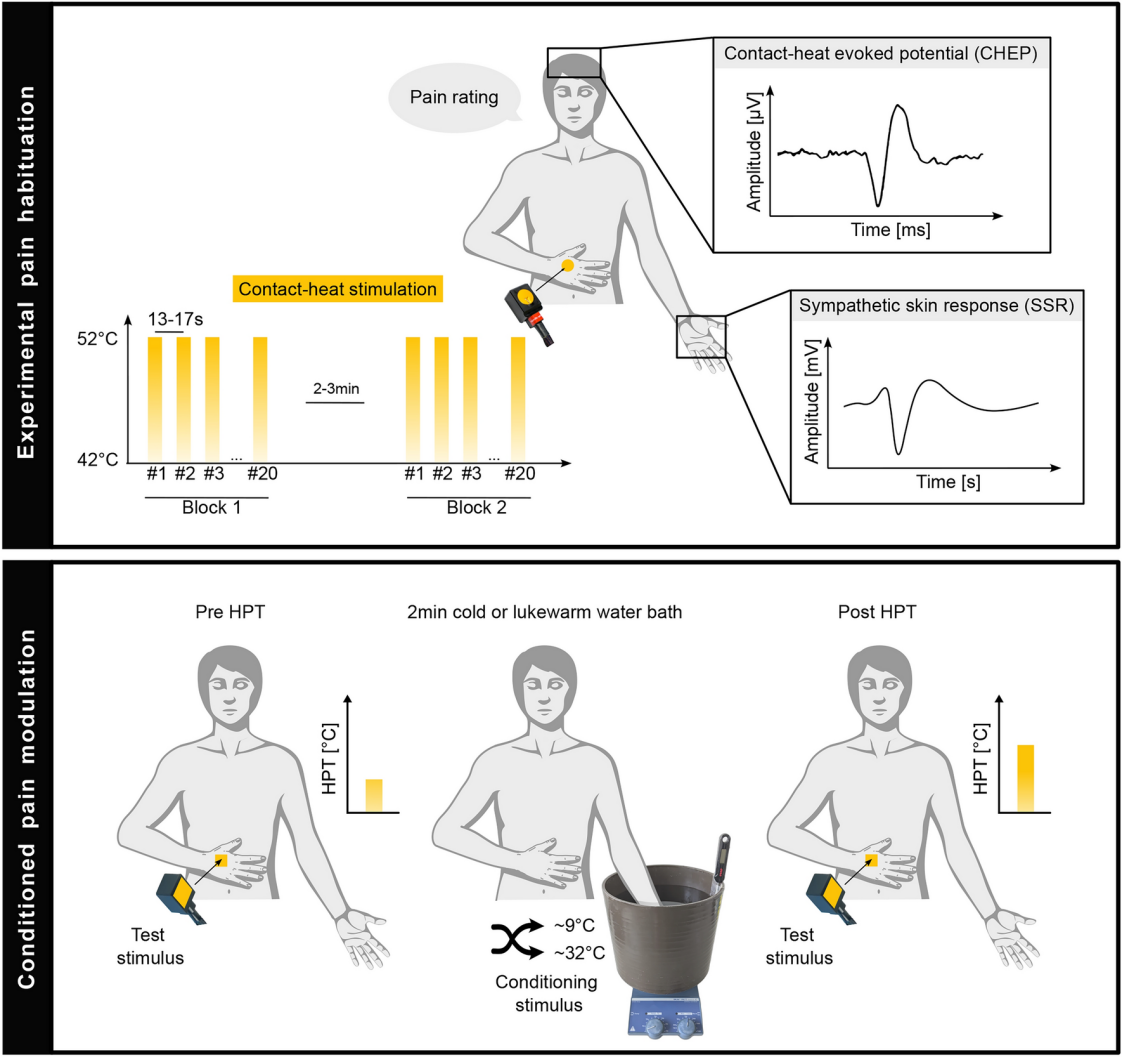
Study design. Experimental pain habituation (upper box) was investigated between two blocks of 20 repetitive contact-heat stimuli while assessing pain ratings, contact-heat evoked potentials (CHEPs), and sympathetic skin responses (SSRs). A sequential conditioned pain modulation paradigm (lower box) was investigated using heat pain threshold (HPT) as test stimulus and a cold or lukewarm water bath as conditioning stimulus (randomized order). Icons were used from BioRender.com.

The participants’ dorsal hand was stimulated with a contact-heat thermode (Pathway model CHEPS, Medoc, Ramat Yishai, Israel) with a heating and cooling rate of 70°/s and 40°/s, respectively. The thermode diameter measured 27 mm. The baseline temperature was set at 42 °C and the destination temperature was set at 52 °C^24^. The stimulation duration was 393 ms, meaning the thermode temperature returned to baseline as soon as the destination temperature was reached. Two stimulation blocks of 15–20 contact-heat stimuli were delivered. The number of stimuli in each block was determined based on the investigator’s real-time assessment, which included monitoring for blink artifacts and alpha wave occurrences. The investigator aimed to ensure a minimum of 15 artifact-free trials. The inter-stimulus-interval was randomized between 13 and 17 s to minimize anticipation^25,26^ and a break of 2-3 min was conducted between the two stimulation blocks (Fig. 1). To minimize peripheral adaptation, the thermode was slightly moved after each contact-heat stimulus within the tested area^27^. After each stimulus, the participant rated the perceived pain following the contact-heat stimulation using a numeric rating scale (NRS) ranging from 0 (no pain) to 10 (maximum pain tolerable). For the analysis, the first 15 pain ratings were averaged. Time locked contact-heat evoked potentials (CHEPs) and sympathetic skin responses (SSRs) were recorded simultaneously.

#### CHEP acquisition

The participants were lying in a comfortable position in a quiet and temperature-controlled room with their gaze fixated on a point. The electroencephalogram (EEG) was recorded from the vertex (Cz) with reference linked to earlobes (A1-A2) in accordance with the international 10–20 system^28^. Additionally, a wet wristband was placed around the volar forearm of the stimulated side and served as the ground. Skin prep gel (Nuprep®, Weaver and Company, Aurora, CO) was used to scrub the recording locations and ethanol 96% (Softasept® N, B. Braun Medical AG, Switzerland) was used to degrease the area afterwards. Single cup electrodes (9 mm Ag/AgCl cup electrodes) were filled with adhesive conductance paste (Elefix, Nihon Kohden Europe GmbH, Germany) and attached to the recording locations. The EEG was measured in the timeframe of 0.5 s pre- to 1 s post-stimulus trigger by a customized LabVIEW software (V2.6.1. CHEP, ALEA Solutions, Zurich, Switzerland) and sampled at 2000 Hz. The signal was pre-amplified (20′000x) and band-pass filtered within 0.5-30 Hz. EEG trials contaminated with eye movement artifacts (recorded by electrooculography using two surface electrodes placed above and below the eye (Ambu BlueSensor NF, Ambu A/S, Ballerup, Denmark)) were identified by two independent investigators and removed offline. Additionally, EEG trials overlaid with alpha waves were discarded from analysis. A minimum of 10 artifact-free trials for each block were averaged and baseline corrected. Both averaged blocks always included the same number of trials.

The first negative and positive peak of the vertex EEG are commonly labeled as N2 and P2, respectively^29^. The detection of the N2P2 waveform of the averaged CHEPs was performed by a custom-made semi-automated algorithm using RStudio (R version 4.1.2 for Windows). To evaluate the signals’ quality, the signal-to-noise ratio (SNR) was calculated as described previously (see Eq. 1 below)^30^. “S” (signal) corresponds to the root mean square of the EEG signal within the time window of 200-700 ms post-stimulus, “N” (noise) to the root mean square of the 500 ms pre-stimulus window. The time window for the signal was chosen based on previous studies collecting normative data of CHEP N2 and P2 latency for upper stimulations.^31,32^ CHEPs with a SNR below 3 dB were considered abolished and were assigned an amplitude of 0 µV^33^. These values of 0 µV were kept for the analysis as they potentially indicate complete CHEP habituation. If the SNR was above 3 dB, the P2 peak was labeled as the maximum positive deflection within the expected time window (200-700 ms post-trigger) exceeding 2SD of the noise signal (500 ms pre-trigger time window). The N2 peak was labeled as the maximum negative deflection prior to the P2 peak exceeding 2SD of the noise signal. If the peaks did not exceed 2SD, an amplitude of 0 µV was assigned for the N2 and P2 peak separately. Accurate labeling of the CHEP amplitude was ensured by two independent investigators in a blinded manner. Unclear instances were reevaluated by the investigators in a consensus meeting.

1 $$SNR = 20*log_{10} \left( \frac{S}{N} \right)$$

#### SSR acquisition

Palmar SSRs elicited by contact-heat stimuli were recorded from the palm contralateral to the stimulated side (Fig. 1). The recording sites were prepared by abrasive scrubbing using Red Dot™ Trace Prep sandpaper tape (3 M, United States), followed by degreasing with 96% ethanol (Softasept® N, B. Braun Medical AG, Switzerland). SSR signals were captured using surface electrodes (Ambu® BlueSensor NF, Ballerup, Denmark), with the active electrode positioned on the palm and the reference electrode on the dorsum of the hand. A wet wrist band was fixated around the volar forearm as the ground. To maintain a skin temperature above 32 °C during the experimental procedure, the skin of the recording site was heated to 32 °C using heating lamps if necessary, as SSR latencies and amplitudes have been shown to be influenced by skin temperature^34^. SSR measurements were acquired within a time frame of 1 s prior to 9 s post-stimulus trigger, utilizing a custom-designed LabVIEW software (V2.6.1. CHEP, ALEA Solutions, Zurich, Switzerland). The data were sampled at a rate of 2000 Hz. SSRs were assessed with a signal amplification by a factor of 20,000 and a subsequent bandpass filtering in the frequency range of 0.1–12 Hz.

A semi-automated algorithm, implemented in R, was used for the determination of SSR amplitudes. In brief, manual identification of the first deflection from baseline (onset) and baseline recovery (offset) of each SSR was performed, and subsequently, the algorithm extracted the maximum peak-to-peak deflection, representing the SSR amplitude, within the designated time window (onset to offset). The onset was required to be at least 1 s post-stimulus, in alignment with prior research demonstrating that SSRs recorded at the hand following noxious heat typically occur between 1.3 and 2.2 s.^35,36^ To ensure precise labelling of SSR amplitudes, two independent investigators evaluated the peak-labelling in a blinded manner. In instances where SSR amplitudes were absent, characterized by flat lines, a value of 0 mV was assigned. As for CHEPs, values of 0 mV were kept for the analysis as they potentially indicate complete SSR habituation. SSRs exhibiting artifacts, such as non-time-locked responses, were assigned a missing amplitude (N/A). A minimum of 10 artifact-free trials were averaged for both experimental blocks, ensuring equal representation of trials in both averaged blocks.

#### Habituation index

For a quantitative assessment of pain habituation, a habituation index for each readout (i.e., pain rating, CHEP and SSR) was calculated and defined as the relative (%) change from the first to the second stimulation block and calculated as follows (Eq. 2):

2 $$Habituation index=\frac{({Block}_{2}-{Block}_{1})}{{Block}_{1}}*100$$

A negative index indicates habituation and a positive index indicates sensitization. In case the first stimulation block had a pain rating of NRS 0 or a CHEP/SSR amplitude of 0V, no habituation index could be calculated, as there was no observable physiological response which could habituate. These participants were excluded from further analysis. The maximum and minimum habituation index was restricted to 100% in the positive and negative direction, respectively.

### Conditioned pain modulation

Two different sequential CPM paradigms were performed in a randomized order (cold water first N = 17, lukewarm water first N = 28) with a break of 10 min between (Fig. 1). During one paradigm, the conditioning stimulus was a cold water bath (9 ± 0.5 °C) and during the other paradigm, the conditioning stimulus was a lukewarm water bath (32 ± 0.5 °C). A thermometer (digital bottle thermometer, Reer, Germany) was used to ensure accurate water temperature. Both water baths were circulating enabled by a magnetic stir (IKA® RET basic C, Huber & Co. AG, Switzerland). The lukewarm water bath was performed to control for repeated measures effect of the test stimulus. Hence, no painful stimulus was present during the lukewarm water bath condition, ensuring that any observed changes in HPT were likely due to peripheral adaptation or sensitization rather than conditioned pain modulation. The conditioning stimulus was applied for 2 min, with participants rating their perceived pain in response to the conditioning stimulus on an NRS from 0 to 10 at the end of the 2 min. The test stimulus was applied on the dorsum of the same hand as the pain habituation paradigm. The test stimulus consisted of the assessment of one HPT before immersion and after withdrawing of the contralateral hand from the water bath (i.e., conditioning stimulus). A heat stimulus was chosen as test stimulus to remain in the same modality as in the pain habituation paradigm. The HPT was performed using the Medoc Pathway System (Pathway model ATS, Medoc, Ramat Yishai, Israel). The thermode measured 30 × 30 mm and the baseline temperature was set at 32 °C. The heating ramp of the thermode was 1 °C/s and the participant was asked to click a button as soon as they felt a second quality in addition to warmth or heat, such as “burning”, “stinging”, “drilling”, or “aching”, in accordance with the instructions provided by the DFNS^23^.

#### CPM effect

The CPM effect was defined as the relative (%) increase or decrease from the pre to post HPT and calculated as follows (Eq. 2):

3 $$CPM effect=\frac{({Pre}_{HPT}-{Post}_{HPT})}{{Post}_{HPT}}*100$$

A negative CPM effect indicated inhibition and a positive CPM effect indicated facilitation. In order to calculate the 'true’ CPM effect (accounting for repeated measures effect of the test stimulus^37–39^), the CPM effect of the lukewarm control water bath was subtracted from the CPM effect of the cold water bath.

### Statistical analysis

All statistical tests were performed in R statistical software (R version 4.1.2 for Windows). Statistical interference was set at an α-level of 0.05, corrected for multiple comparisons by a Benjamini–Hochberg correction. Normality of the data was tested using a Shapiro–Wilk test.

A one-sample t-test against zero (two-sided) was performed to investigate whether there was significant pain habituation or CPM effect. To meet the statistical analysis criteria, outliers with a standard deviation greater than two were removed.

For the correlation analyses, Pearson and Spearman correlations were used for normally and non-normally distributed data, respectively. Influential cases were identified by inspecting the correlation’s residuals vs fitted plot, normal q-q plot and Cook’s distance using the lm() function. Outliers which were consistently reported in the residuals vs fitted plot, normal q-q plot and Cook’s distance were removed from the analysis. Because the main goal was to explain the variability of pain habituation, all correlations were performed in relation to the habituation indices. Firstly, the habituation indices of the three different readouts were correlated with each other. Next, the habituation indices were correlated with the uncorrected CPM effect (cold-water bath) and the ‘true’ CPM effect (corrected values). Further, correlations between the habituation indices and the participants’ age as well as baseline pain sensitivity (i.e., HPT of first visit) were evaluated. Additionally, the HADS and PCS scores were correlated with the habituation indices to examine a potential psychological contribution to the observed variability. Finally, a potential sex influence on pain habituation was investigated by dividing participants into two groups depending on their sex. These groups were then compared in terms of habituation indices using an unpaired t-test.

## Results

### Descriptive statistics of the pain habituation index and CPM effect

In total, 45 participants were included in this secondary analysis comprising 22 female and 23 male participants. The mean age was 49.4y, ranging from 21 to 75y. The raw values of pain ratings, CHEPs and SSRs parameters after the first and second block of contact-heat stimulation are provided in Table 1. Additionally, Table 1 illustrates the HPT assessed pre and post immersion of the contralateral hand into the cold (9 °C) and lukewarm control (32 °C) water bath.

**Table 1:** Raw values of readouts for pain habituation and conditioned pain modulation.

Experimental pain habituation		
	Block 1	Block 2
Pain rating [NRS]	4.1 (2.0, 0.8–9.1)	3.6 (2.0, 0.6–8.9)***
CHEP [μV]	32.0 (15.2, 0.0–64.9)	26.9 (15.4, 0.0–68.2)***
SSR [mV]	2.8 (2.6, 0.1–12.4)	1.6 (1.7, 0.0–7.9)***

Conditioned pain modulation		
	Pre HPT [°C]	Post HPT [°C]
Cold water batsh	43.3 (3.7, 35.8–51.0)	45.6 (3.5, 37.1–51.0)***
Lukewarm water bath	43.4 (3.5, 35.7–51.0)	45.2 (3.5, 36.7–51.0)***

Data is presented as mean (SD, range). Abbreviations: CHEP: Contact-heat evoked potential; HPT: Heat pain threshold; NRS: Numeric rating scale; SSR: Sympathetic skin response.

*** *p* < 0.001 (tested as percentage change from zero, (one-sample t-test)).

**Fig. 2:**
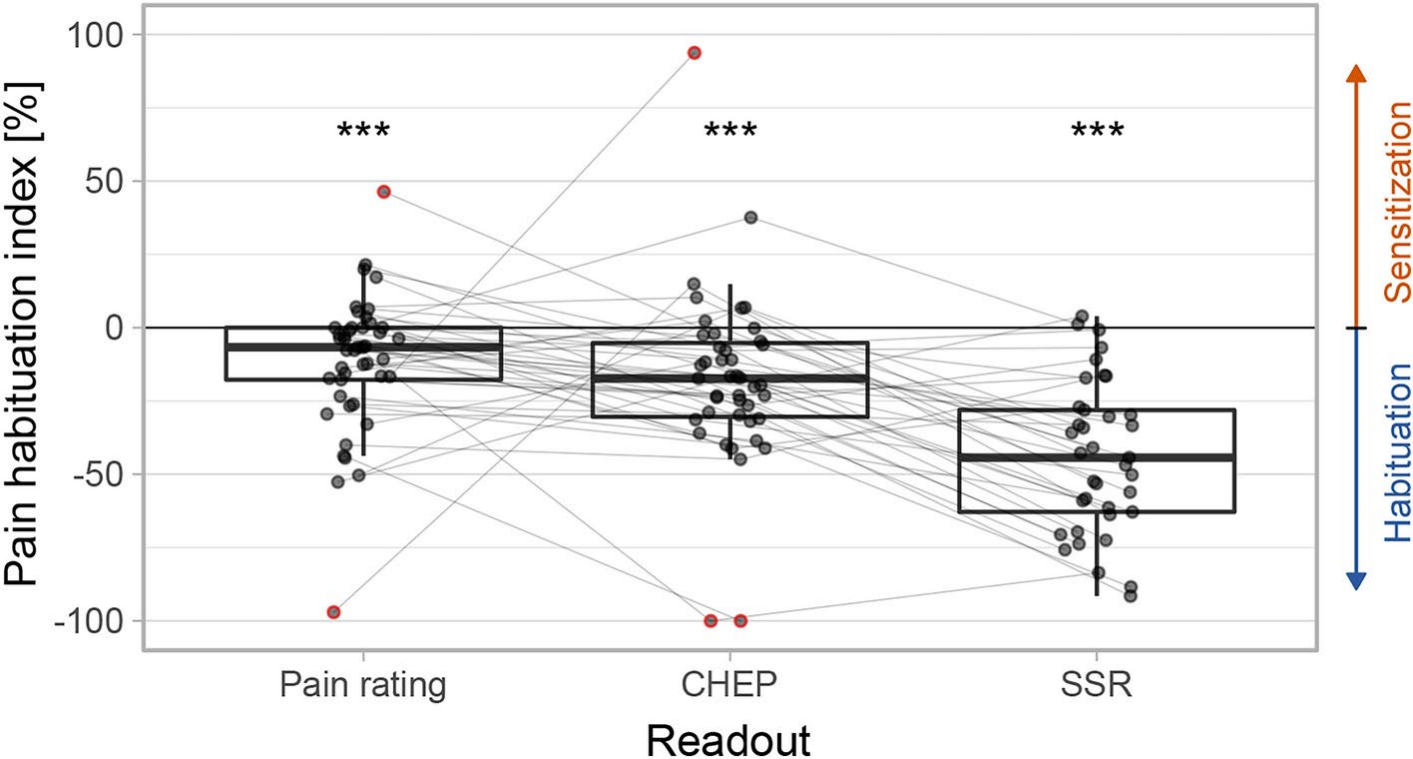
Habituation index of pain ratings (N = 45), contact-heat evoked potentials (CHEPs, N = 43), and sympathetic skin responses (SSRs, N = 37). Outliers are marked red and were removed from statistical analysis to meet the statistical analysis criteria (pain ratings outliers N = 2, CHEP outliers N = 3). ***p < 0.001.

**Fig. 3:**
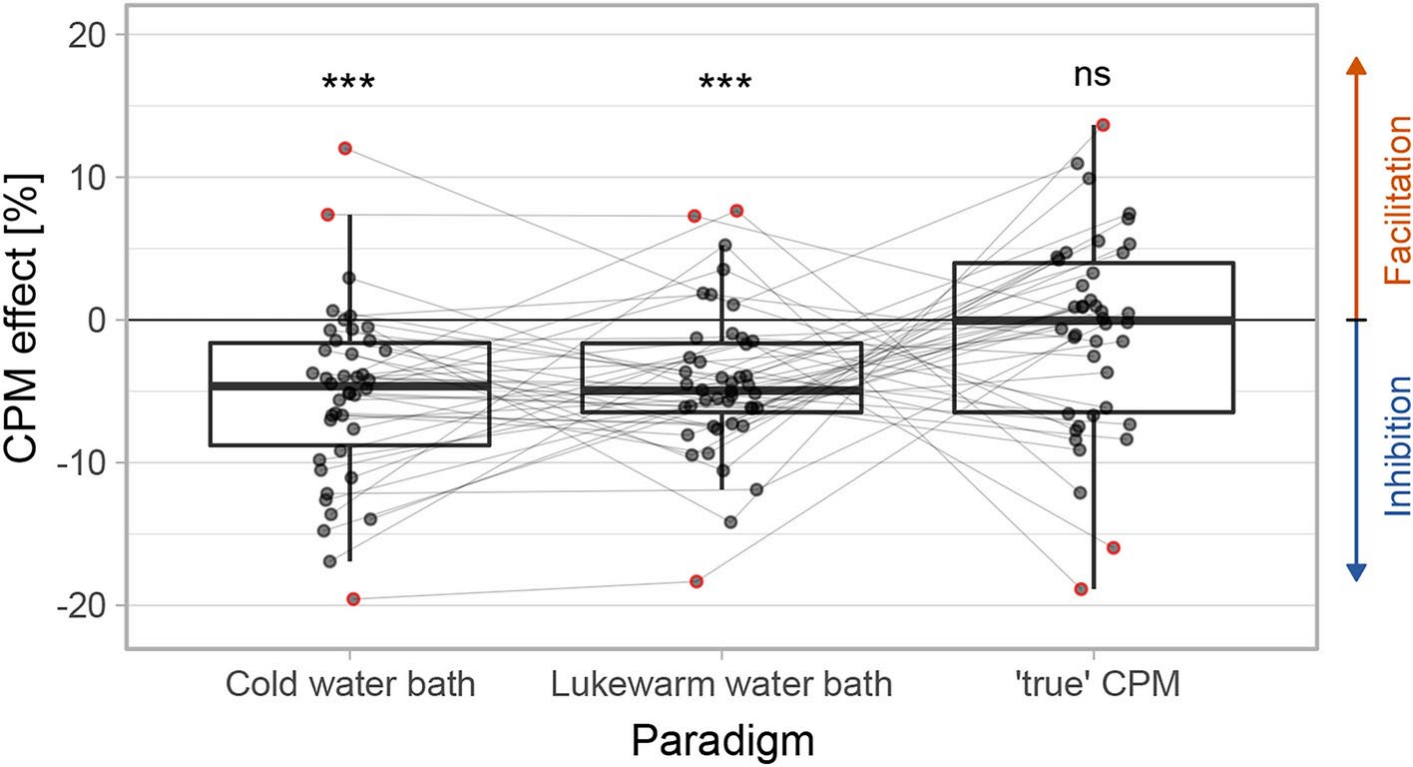
Conditioned pain modulation (CPM) effect. Illustrated are the cold water bath paradigm (i.e., uncorrected CPM effect, N = 42), the lukewarm water bath paradigm (N = 44) and the ‘true’ CPM effect (corrected, N = 42). Outliers are marked red and were removed from statistical analysis to meet the statistical analysis criteria (pain ratings outliers N = 3, CHEP outliers N = 3, SSR outliers N = 3). ***p < 0.001, ns *p* > 0.05.

Figure 2 illustrates the habituation indices between the two stimulation blocks for pain ratings, CHEPs, and SSRs. For CHEPs only, two participants were excluded due to an abolished CHEP in the first stimulation block. Regarding SSRs, eight participants were excluded, because they had too few artifact-free trials (< 10 trials) in one of the two stimulation blocks. To get an impression of the progression of the assessed readouts, supplementary Figure S1 illustrates the single trials over the two stimulation blocks for pain ratings an SSRs. For CHEPs, single trials are not illustrated because they typically have low SNR. There was a significant habituation between the two stimulation blocks regardless of the readout (pain ratings: t(42) = − 4.1, *p* < 0.001; CHEPs: t(39) = -5.8, *p* < 0.001; SSRs: t(36) =  − 10.4, *p* < 0.001). Moreover, the CPM effect is illustrated in Fig. 3 for the cold water bath paradigm only, the lukewarm water bath paradigm only, and the corrected values (‘true’ CPM effect). Three participants did not tolerate the cold water bath for two minutes and were excluded. The average perceived pain intensity of the cold water bath was 7.5 ± 2.0 NRS. There was a significant increase in HPT for the cold water bath paradigm (i.e., uncorrected CPM effect) (t(38) = − 7.4, *p* < 0.001) as well as the lukewarm water bath paradigm (t(40) = − 7.5, *p* < 0.001), but not when investigating the ‘true’ CPM effect (corrected for repeated measures) (t(38) = − 0.5, *p* = 0.718).

### Associations of pain habituation with CPM and biopsychological factors

**Fig. 4:**
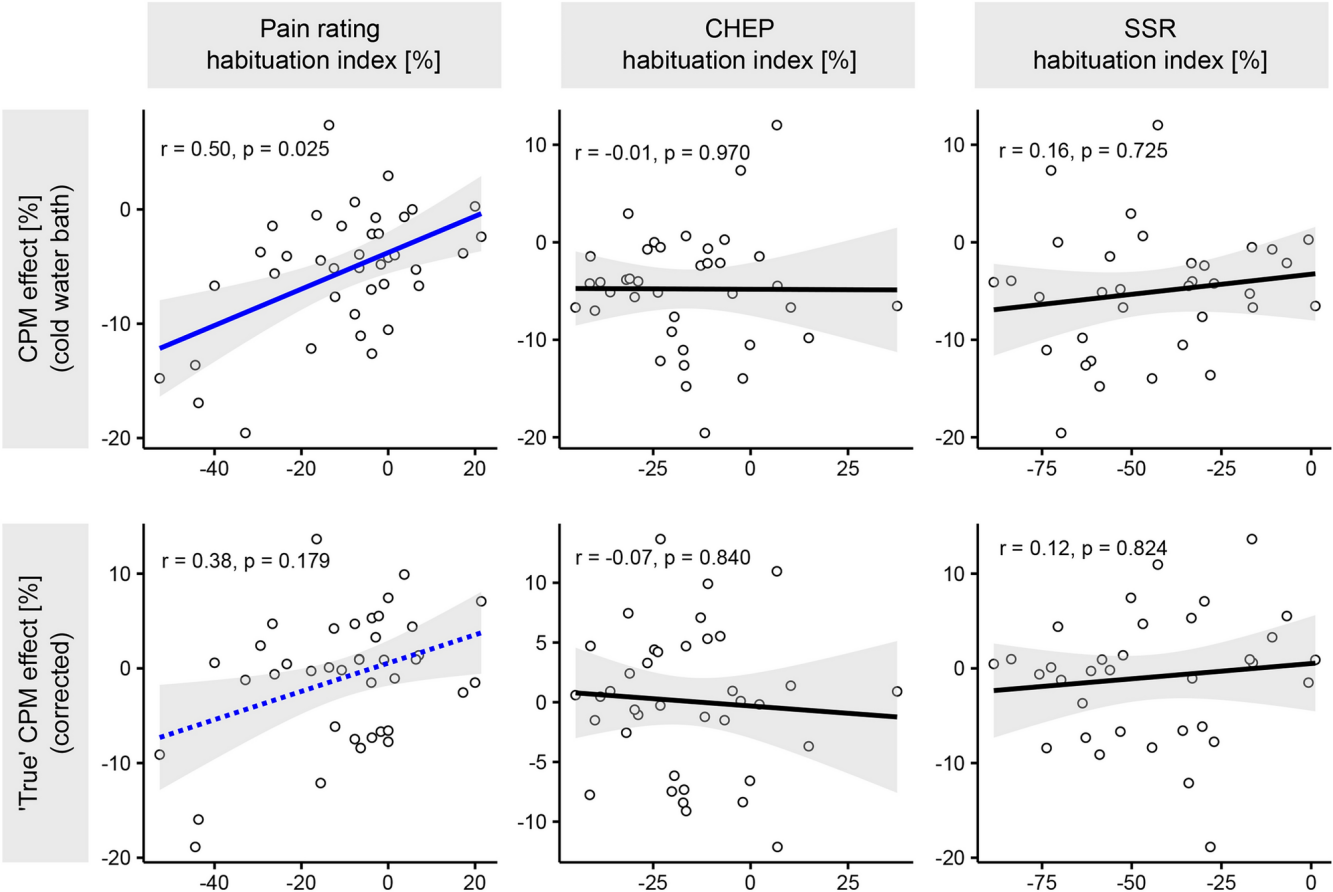
Correlations between the habituation indices and conditioned pain modulation (CPM) effects. Significant correlations are illustrated by a blue regression line and non-significant correlations by a black regression line. The dashed blue line indicates a significant correlation prior to multiple comparison correction. CHEP: Contact-heat evoked potential; SSR: Sympathetic skin response.

**Fig. 5:**
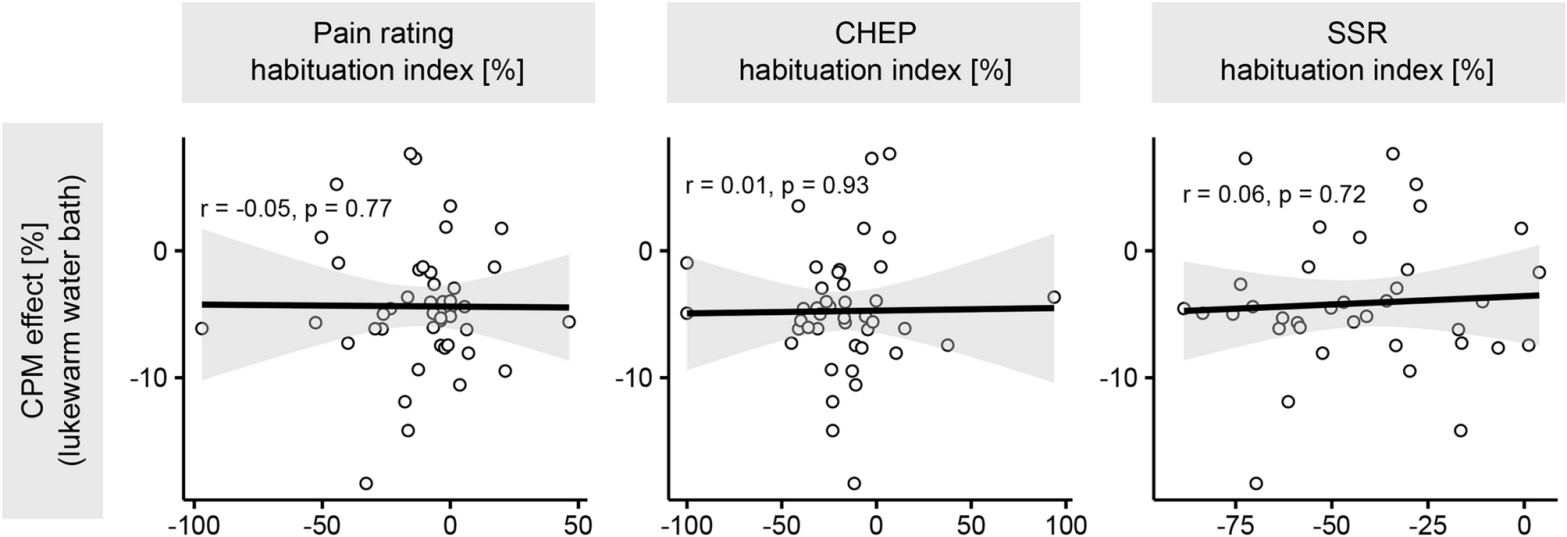
Correlations between the habituation indices and conditioned pain modulation (CPM) effects after the lukewarm water bath. Non-significant correlations are illustrated by a black regression line. CHEP: Contact-heat evoked potential; SSR: Sympathetic skin response.

A detailed summary of the correlation analyses performed can be found in the supplementary Table S1. The different habituation indices (i.e., pain ratings, CHEPs, and SSRs) did not correlate with each other (all *p*’s > 0.05). Interestingly, the uncorrected CPM effect (cold water bath) correlated with the habituation index of pain ratings (Fig. 4). This correlation was still observed for the ‘true’ CPM effect but lost its significance after correcting for multiple comparisons (Fig. 4). All remaining correlations between habituation indices and CPM effects were not significant (Fig. 4). Because there was generally no significant ‘true’ CPM effect (Fig. 3), an additional exploratory sub-analysis was conducted where the CPM effect during the lukewarm water bath only was correlated with the pain habituation index. By doing so, we could further investigate whether the observed correlation between CPM effect (cold water bath only) and subjective pain habituation index was mainly driven by peripheral adaptation/sensitization processes due to repeated testing of the HPT (test stimulus). However, the correlation between the CPM effect during the lukewarm water bath only and subjective pain habituation was not significant (r = − 0.05, *p* = 0.77, Fig. 5). Additionally, neither the participants’ HPT assessed in the first visit (baseline pain sensitivity) nor age correlated with the habituation indices (all *p*’s > 0.05). There were no differences in pain habituation between male and female participants (all *p*’s > 0.05, supplementary Table S2). Finally, the habituation indices did not correlate with the HADS (mean = 5.1, SD = 4.0, range = 0 to 21, N moderate anxiety = 2, N high anxiety = 1, N moderate/high depression = 0) or PCS scores (mean = 5.6, SD = 6.9, range = 0 to 23, N clinically significant catastrophizing = 0) (all *p* > 0.05).

## Discussion

The aim of this study was to investigate whether inter-individual differences in subjective (i.e., pain ratings) and objective readouts (i.e., CHEPs and SSRs) of pain habituation were related to CPM effects and biopsychological factors. Subjective pain habituation correlated positively with the CPM effect when using a cold water bath as the conditioning stimulus. However, after correcting for multiple comparisons the magnitude of pain habituation did not correlate with the ‘true’ CPM effect, i.e., when repeated testing was corrected for by subtracting the lukewarm from the cold water bath condition. Biological and psychological factors did not correlate with pain habituation regardless of the readout.

This study incorporated subjective and objective readouts of pain habituation to gain a more comprehensive understanding of pain habituation processes. Interestingly and in line with previous research^12^, the three different habituation indices, i.e., pain ratings, CHEPs, and SSRs, did not correlate^12^ or show the same modulation pattern^5,11^. This may be explained by the heterogenous origins (different neural substrates involved) of pain rating, CHEPs and SSRs. In more detail, two distinct neural pathways are pivotal in pain perception:^40^ (1) the sensory-discriminative; and (2) the affective-motivational pathway. The sensory-discriminative pathway primarily encodes the location, intensity, and quality of pain, involving brain regions such as the somatosensory cortex and thalamus. In contrast, the affective-motivational pathway encodes the emotional and motivational aspects of pain, e.g., pain unpleasantness, with crucial involvement from areas such as the ACC and the limbic system. Consequently, pain intensity ratings and habituation thereof might be predominantly associated with the sensory-discriminative pathway, while pain unpleasantness is more closely tied to the affective-motivational pathway. In contrast, pain-related evoked potentials recorded from the vertex such as the CHEPs in the present study reflect ACC activation^41,42^, highlighting their origin in the affective-motivational pathway. Finally, SSRs incorporate additional spinal and bulbar processes^13^, while being highly influenced by affective-motivational components as reviewed by Beissner and colleagues^43^. Therefore, while subjective pain ratings and objective CHEPs/SSRs may share certain overlapping neural substrates, fundamental differences in their neural origins potentially explain the lack of correlation between all three readouts in the present study. Based on the hypothesis that subjective and objective responses to noxious stimuli reflect activities within distinct neural substrates, previous research in chronic pain focusing only on one particular pain habituation readout might have overlooked changes across different nociceptive pathways. Therefore, the use of multiple pain habituation readouts might uncover subclinical alterations within otherwise overlooked aspects of the central nervous system.

Previous research by Eitner and colleagues^14^ highlighted that CPM effects, assessed through both subjective pain ratings and objective neurophysiological readouts, cannot be fully accounted for by pain habituation alone (hence, different underlying mechanism). Our study, focused on examining how differences in descending pain modulation (i.e., CPM effects) might explain the variability in individual pain habituation responses. Understanding this relationship is particularly relevant for benchmarking deficient pain habituation as a potential indicator of a pronociceptive shift in pain modulation^2,44,45^, which is of interest to establish targeted pain-treatment strategies. In line with findings of Treister and colleagues^10^, subjective pain habituation (i.e., habituation of pain ratings) correlated positively with the CPM effect. Specifically, participants who displayed more pronounced habituation in pain ratings showed greater inhibitory capacity during the CPM paradigm. This was, however, not true for the objective habituation readouts, namely CHEPs and SSRs habituation. The observed correlation with subjective pain habituation alone could be attributed to the fact that both subjective pain habituation and CPM effects were assessed using subjective readouts likely reflecting nociceptive processing within similar neural networks, i.e., sensory-discriminative, as discussed in Section "Descriptive statistics of the pain habituation index and CPM effect". Hence, further studies should investigate whether CHEP and SSR habituation potentially correlates with CPM effects assessed by neurophysiological (CHEP) and autonomic (SSR) readouts.

When the CPM effect was adjusted by additionally using a control paradigm involving lukewarm water, the correlation with subjective pain habituation was no longer significant. The control CPM paradigm was introduced to enable correction for repeated measure effects such as peripheral adaptation or sensitization processes. Importantly, the HPT was increased to a similar amount during both the cold and the lukewarm water bath (mean HPT increase (CPM effect of ~ 5%)). Therefore, there was no ‘true’ CPM effect (Fig. 3). This observation has been previously explained by the fact that even non-noxious stimuli such as the lukewarm water bath can induce endogenous inhibitory modulation of test stimuli^10^. Animal studies, however, did not confirm the concept of 'non-noxious induced endogenous inhibition’^46^. We would rather argue that the observed increase in HPT might be attributed to peripheral processes. Hence, HPT might not be the optimal test stimulus to assess CPM effects. However, it is important to note the broad variability observed in the ‘true’ CPM effects among the participants even when corrected for peripheral adaptation. This variability was crucial to our study, as it allowed an investigation into whether individuals with more pronounced pain habituation also demonstrated enhanced ‘true’ CPM effects. Thus, while the CPM effects after a cold water bath could be due to both peripheral and central pain modulatory mechanisms, the “true” CPM effect may lack these peripheral aspects of pain modulation (i.e., peripheral adaptation). Consequently, the observation that pain habituation only correlated with the CPM effect following a cold water bath suggests that peripheral adaptation might partly contribute to pain habituation as previously reported^27^. This could be due to a residual overlap of stimulated receptors even after slightly moving the contact-heat thermode after each stimulus as performed in the present study. Yet, it should be noted that CPM effects after the lukewarm water bath only, predominantly reflecting peripheral adaptation, did not correlate with the subjective pain habituation index. These findings suggest that the variability in subjective pain habituation is probably due to a combination of both peripheral adaptation and endogenous pain modulation.

Touching upon biological (i.e., age and sex) and psychological factors (i.e., HADS and PCS score) potentially explaining inter-individual differences in pain habituation, we found no relationship with these factors regardless of the pain habituation readout. More specifically on biological/demographic factors, we hypothesized that older participants would present with reduced pain habituation compared to younger participants based on previous findings showing reduced endogenous inhibitory capacity with age^20,47,48^. Contrary to findings reported by Edwards and colleagues^20^, we could not confirm that older participants presented with reduced pain habituation regardless of the habituation readout. Consistent with our findings, a more recent study found no effect of age on pain habituation^49^. Furthermore, sex hormones, such as estrogen, progesterone, and testosterone, have differential effects on pain sensitivity (for review see^50^). For instance, testosterone has been discussed to have predominantly anti-nociceptive and protective effects in both men and women, with lower testosterone levels being associated with chronic pain. The anti-nociceptive effects of testosterone could have resulted in increased pain habituation in males; however, the hypothesis of pain habituation being more pronounced in males was not confirmed in the present study. Previous studies on sex differences in pain habituation have yielded inconsistent findings. Ginzburg and colleagues^21^ found that male participants are more likely to show complete habituation than female participants. Two other studies reported no significant differences between sexes in pain habituation^10,27^, while another two studies found that women exhibited greater pain habituation compared to men^51,52^. However, as reviewed by van der Miesen and colleagues, these discrepancies may be due to the use of individually adapted stimulation intensities between the two sexes (lower stimulation intensities in women), rather than inherent biological differences between men and women^49^.

Concerning psychological factors (i.e., anxiety, depression, and pain catastrophizing), it is well established that there is a bidirectional relationship between these factors and pain sensitivity as well as pain modulation (for review see^53,54^). For instance, Edwards and colleagues reported reduced habituation in women with increased pain catastrophizing scores^16^. In our cohort of healthy participants, no such correlation was found, potentially due to a floor effect of the HADS and PCS scores in our study sample. Furthermore, Nakamura and colleagues observed that the anxiety state but not the anxiety trait was associated with pain habituation^12^. The absence of a relationship between anxiety (or psychological factors in general) and experimental pain habituation could therefore be attributed to the fact that the HADS primarily evaluates the anxiety trait and not state. Finally, no correlations between pain habituation and biopsychological factors might be detected because either the relationship is not linear or no single factor could explain the observed variability in pain habituation. Rather, there might be a complex interplay between these and other factors.

While this study provides valuable insights into the relationship between two proxies of endogenous pain modulation, it is essential to consider certain limitations. Due to time constrains only two blocks of heat stimulation were applied to assess pain habituation. This may have hindered the establishment a complete habituation pattern. Nevertheless, we were able to observe statistically significant pain habituation for all habituation readouts and are therefore confident that this does not limit the interpretability of our results. Additionally, applying up to 40 stimulations of contact-heat to investigate the habituation of pain ratings^55^, CHEPs^5,27,56–60^, and SSRs^61,62^ is in line with stimulation protocols applied in previous literature. Additionally, the larger study also included other test stimuli (i.e., pressure and consecutive stimuli) during the CPM protocol which might have sensitized the stimulation area. We focused in this study only on heat stimuli as pain habituation was also assessed using noxious contact-heat stimuli. Moreover, the CPM effect might have been influenced by the randomization of the visits and CPM water bath order (lukewarm or cold first). We, however, found no effect of the randomization order on CPM effects (Welch two sample t-test: all *p*’s > 0.05). Lastly, the CPM effect assessed in this study might also have been influenced by segmental effects as the conditioning stimulus was contralaterally, but not extra-segmentally applied. Hence, purely spinal mechanisms might have been involved in the observed CPM effect.

Taken together, the present study found that subjective pain habituation correlated with the uncorrected CPM effect (not corrected for repeated measure effects). This indicates that the inter-individual variability of subjective pain habituation may arise from differences in descending pain modulatory capacities as well as peripheral adaptation. The variability in pain habituation of objective readouts, which potentially reflect affective-motivational pain processing (CHEPs) and additional bulbospinal processes (SSRs), is still unclear. It appears that the variability of CHEP and SSR habituation are not attributable to biological factors such as sex and age, nor can they be explained by psychological factors such as pain catastrophizing or depressive and anxious mood. The fact that there was no association between subjective and objective pain habituation indicates that objective habituation readouts are complementary rather than congruent to subjective habituation readouts and may pose two useful additions to study pain habituation in clinical cohorts.

## Supplementary Information

Supplementary Information.

## Data Availability

Data and programming codes are available upon request.

## References

[1] Treede, R.-D. *et al.* Chronic pain as a symptom or a disease: the IASP classification of chronic pain for the international classification of diseases (ICD-11). *Pain* **160**, 19–27 (2019). 30586067 10.1097/j.pain.0000000000001384

[2] Yarnitsky, D. Role of endogenous pain modulation in chronic pain mechanisms and treatment. *Pain* **156**(Suppl), S24–S31 (2015). 25789433 10.1097/01.j.pain.0000460343.46847.58

[3] Rankin, C. H. *et al.* Habituation revisited: an updated and revised description of the behavioral characteristics of habituation. *Neurobiol. Learn Mem.* **92**, 135–138 (2009). 18854219 10.1016/j.nlm.2008.09.012 PMC2754195

[4] van der Miesen, M. M., Vossen, C. J. & Joosten, E. A. Habituation to pain in patients with chronic pain: clinical implications and future directions. *J. Clin. Med.* **12**(13), 4305 (2023). 37445339 10.3390/jcm12134305 PMC10342770

[5] De Schoenmacker, I., Leu, C., Curt, A. & Hubli, M. Pain-autonomic interaction is a reliable measure of pain habituation in healthy subjects. *Eur. J. Pain* **26**, 1679–1690 (2022). 35671124 10.1002/ejp.1990 PMC9544564

[6] van der Miesen, M. M. *et al.* Habituation to pain: self-report, electroencephalography, and functional magnetic resonance imaging in healthy individuals. A scoping review and future recommendations. *Pain*10.1097/j.pain.00000000000030525 (2023). 37851343 10.1097/j.pain.0000000000003052 PMC10859850

[7] Ibinson, J. W. *et al.* Functional magnetic resonance imaging studies of pain: an investigation of signal decay during and across sessions. *Anesthesiology* **101**, 960–969 (2004). 15448530 10.1097/00000542-200410000-00022

[8] Becerra, L. R. *et al.* Human brain activation under controlled thermal stimulation and habituation to noxious heat: an fMRI study. *Magn. Reson. Med.* **41**, 1044–1057 (1999). 10332889 10.1002/(sici)1522-2594(199905)41:5<1044::aid-mrm25>3.0.co;2-m

[9] Kennedy, D. L., Kemp, H. I., Ridout, D., Yarnitsky, D. & Rice, A. S. C. Reliability of conditioned pain modulation: a systematic review. *Pain*10.1097/j.pain.0000000000000689 (2016). 27559835 10.1097/j.pain.0000000000000689 PMC5228613

[10] Treister, R., Eisenberg, E., Gershon, E., Haddad, M. & Pud, D. Factors affecting - and relationships between-different modes of endogenous pain modulation in healthy volunteers. *Eur. J. Pain* **14**, 608–614 (2010). 19906552 10.1016/j.ejpain.2009.10.005

[11] Breimhorst, M., Hondrich, M., Rebhorn, C., May, A. & Birklein, F. Sensory and sympathetic correlates of heat pain sensitization and habituation in men and women. *Eur. J. Pain (United Kingdom)* **16**, 1281–1292 (2012). 10.1002/j.1532-2149.2012.00133.x 22407985

[12] Nakamura, Y., Donaldson, G. W. & Okifuji, A. Personality, anxiety, and individual variation in psychophysiological habituation and sensitization to painful stimuli. *J. Pain Relief* **3**(3), 1–9 (2014).

[13] Wang, G. H. The galvanic skin reflex; a review of old and recent works from a physiologic point of view. *II. Am. J. Phys. Med.* **37**, 35–57 (1958). 13508858

[14] Eitner, L. *et al.* Conditioned pain modulation using painful cutaneous electrical stimulation or simply habituation?. *Eur. J. Pain (United Kingdom)* **22**, 1281–1290 (2018). 10.1002/ejp.1215 29573038

[15] De Paepe, A. L. & de Williams, A. C. Habituation to pain: a motivational-ethological perspective. *Pain* **160**, 1693–1697 (2019). 31335639 10.1097/j.pain.0000000000001533

[16] Edwards, R. R., Smith, M. T., Stonerock, G. & Haythornthwaite, J. A. Pain-related catastrophizing in healthy women is associated with greater temporal summation of and reduced habituation to thermal pain. *Clin. J. Pain* **22**(8), 730–737 (2006). 16988570 10.1097/01.ajp.0000210914.72794.bc

[17] Bingel, U., Schoell, E., Herken, W., Büchel, C. & May, A. Habituation to painful stimulation involves the antinociceptive system. *Pain* **131**, 21–30 (2007). 17258858 10.1016/j.pain.2006.12.005

[18] Hopper, J. W., Frewen, P. A., van der Kolk, B. A. & Lanius, R. A. Neural correlates of reexperiencing, avoidance, and dissociation in PTSD: symptom dimensions and emotion dysregulation in responses to script-driven trauma imagery. *J. Trauma Stress* **20**, 713–725 (2007). 17955540 10.1002/jts.20284

[19] Rainville, P., Duncan, G. H., Price, D. D., Carrier, B. & Bushnell, M. C. Pain affect encoded in human anterior cingulate but not somatosensory cortex. *Science* **277**, 968–971 (1997). 9252330 10.1126/science.277.5328.968

[20] Edwards, R. R. & Fillingim, R. B. Effects of age on temporal summation and habituation of thermal pain: clinical relevance in healthy older and younger adults. *J. Pain* **2**, 307–317 (2001). 14622810 10.1054/jpai.2001.25525

[21] Ginzburg, K. *et al.* Body awareness and pain habituation: the role of orientation towards somatic signals. *J. Behav. Med.* **38**, 876–885 (2015). 26341355 10.1007/s10865-015-9676-8

[22] Sullivan, M. J. L. & Bishop, S. R. The pain catastrophizing scale: development and validation. *J. Physiother.* **7**, 524–332 (1995).

[23] Rolke, R. *et al.* Quantitative sensory testing: a comprehensive protocol for clinical trials. *Eur. J. Pain* **10**, 77 (2006). 16291301 10.1016/j.ejpain.2005.02.003

[24] Kramer, J. L., Haefeli, J., Jutzeler, C. R., Steeves, J. D. & Curt, A. Improving the acquisition of nociceptive evoked potentials without causing more pain. *Pain* **154**(2), 235–41 (2013). 23218174 10.1016/j.pain.2012.10.027

[25] Oka, S. *et al.* Predictability of painful stimulation modulates subjective and physiological responses. *J. Pain* **11**, 239–246 (2010). 19853519 10.1016/j.jpain.2009.07.009

[26] Baumgärtner, U., Greffrath, W. & Treede, R. D. Contact heat and cold, mechanical, electrical and chemical stimuli to elicit small fiber-evoked potentials: merits and limitations for basic science and clinical use. *Neurophysiol. Clin.* **42**, 267–280 (2012). 23040698 10.1016/j.neucli.2012.06.002

[27] Greffrath, W., Baumgärtner, U. & Treede, R. D. Peripheral and central components of habituation of heat pain perception and evoked potentials in humans. *Pain* **132**, 301–311 (2007). 17533117 10.1016/j.pain.2007.04.026

[28] Klem, G. H., Lüders, H. O., Jasper, H. H. & Elger, C. The ten-twenty electrode system of the international federation. *Electroencephalogr. Clin. Neurophysiol.* **10**, 371–375 (1999). 10590970

[29] García-larrea, L. *et al.* Association and dissociation between laser-evoked potentials and pain perception. *Neuroreport* **8**, 3785–3789 (1997). 9427371 10.1097/00001756-199712010-00026

[30] De Schoenmacker, I., Archibald, J., Kramer, J. L. K. & Hubli, M. Improved acquisition of contact heat evoked potentials with increased heating ramp. *Sci. Rep.* **12**, 925 (2022). 35042939 10.1038/s41598-022-04867-y PMC8766469

[31] Granovsky, Y. *et al.* Normative data for Aδ contact heat evoked potentials in adult population: a multicenter study. *Pain* **157**, 1156–1163 (2016). 26907092 10.1097/j.pain.0000000000000495

[32] Jutzeler, C. R., Rosner, J., Rinert, J., Kramer, J. L. K. & Curt, A. Normative data for the segmental acquisition of contact heat evoked potentials in cervical dermatomes. *Nat. Publish. Group*10.1038/srep34660 (2016). 10.1038/srep34660 PMC5052572 27708413

[33] De Schoenmacker, I. *et al.* An intensity matched comparison of laser- and contact heat evoked potentials. *Sci. Rep.* **11**, 1–12 (2021). 33767259 10.1038/s41598-021-85819-w PMC7994633

[34] Deltombe, T., Hanson, P., Jamart, J. & Clérin, M. The influence of skin temperature on latency and amplitude of the sympathetic skin response in normal subjects. *Muscle Nerve* **21**, 34–39 (1998). 9427221 10.1002/(sici)1097-4598(199801)21:1<34::aid-mus5>3.0.co;2-h

[35] Cervera, A., Veciana, M. & Valls-Solé, J. Sympathetic sudomotor skin responses induced by laser stimuli in normal human subjects. *Neurosci. Lett.* **334**, 115–118 (2002). 12435485 10.1016/s0304-3940(02)01117-5

[36] Rossi, P. *et al.* Sympathetic skin response evoked by laser skin stimulation. *Funct. Neurol.* **17**, 129–132 (2002). 12549717

[37] Lütolf, R. *et al.* Anti- and Pro-Nociceptive mechanisms in neuropathic pain after human spinal cord injury. *Eur. J. Pain*10.1002/ejp.2029 (2022). 36000307 10.1002/ejp.2029 PMC9826499

[38] Granot, M. *et al.* Determinants of endogenous analgesia magnitude in a diffuse noxious inhibitory control (DNIC) paradigm: do conditioning stimulus painfulness, gender and personality variables matter?. *Pain* **136**, 142–149 (2008). 17720319 10.1016/j.pain.2007.06.029

[39] Kennedy, D. L., Kemp, H. I., Wu, C., Ridout, D. A. & Rice, A. S. C. Determining real change in conditioned pain modulation: a repeated measures study in healthy volunteers. *J. Pain* **21**, 708–721 (2020). 31715262 10.1016/j.jpain.2019.09.010

[40] Schaible, H. G. Peripheral and central mechanisms of pain generation. *Handbook Exp. Pharmacol.* **177**, 3–28 (2007). 10.1007/978-3-540-33823-9_1 17087118

[41] Vogt, B. A. Pain and emotion interactions in subregions of the cingulate gyrus. *Nat. Rev. Neurosci.* **6**, 533–544 (2005). 15995724 10.1038/nrn1704 PMC2659949

[42] Garcia-Larrea, L., Frot, M. & Valeriani, M. Brain generators of laser-evoked potentials: from dipoles to functional significance. *Neurophysiol. Clin.* **33**, 279–292 (2003). 14678842 10.1016/j.neucli.2003.10.008

[43] Beissner, F., Meissner, K., Bär, K. J. & Napadow, V. The autonomic brain: An activation likelihood estimation meta-analysis for central processing of autonomic function. *J. Neurosci.* **33**, 10503–10511 (2013). 23785162 10.1523/JNEUROSCI.1103-13.2013 PMC3685840

[44] Nijs, J. *et al.* Applying modern pain neuroscience in clinical practice: criteria for the classification of central sensitization pain. *Pain Phys.* **17**, 447–457 (2014). 25247901

[45] Arendt-Nielsen, L. *et al.* Assessment and manifestation of central sensitisation across different chronic pain conditions. *Eur. J. Pain* **22**, 216–241 (2018). 29105941 10.1002/ejp.1140

[46] Le Bars, D., Villanueva, L., Bouhassira, D. & Willer, J. C. Diffuse noxious inhibitory controls (DNIC) in animals and in man. *Patologicheskaia Fiziologiia I Eksperimental’naia Terapiia* **1**(4), 55–65 (1992). 1303506

[47] Edwards, R. R., Fillingim, R. B. & Ness, T. J. Age-related differences in endogenous pain modulation: a comparison of diffuse noxious inhibitory controls in healthy older and younger adults. *Pain* **101**, 155–165 (2003). 12507710 10.1016/s0304-3959(02)00324-x

[48] Washington, L. L., Gibson, S. J. & Helme, R. D. Age-related differences in the endogenous analgesic response to repeated cold water immersion in human volunteers. *Pain* **89**, 89–96 (2000). 11113297 10.1016/S0304-3959(00)00352-3

[49] van der Miesen, M. M. *et al.* Habituation to pain: self-report, electroencephalography, and functional magnetic resonance imaging in healthy individuals. A scoping review and future recommendations. *Pain*10.1097/j.pain.0000000000003052 (2023). 37851343 10.1097/j.pain.0000000000003052 PMC10859850

[50] Cairns, B. E. & Gazerani, P. Sex-related differences in pain. *Maturitas* **63**, 292–296 (2009). 19595525 10.1016/j.maturitas.2009.06.004

[51] Defrin, R., Pope, G. & Davis, K. D. Interactions between spatial summation, 2-point discrimination and habituation of heat pain. *Eur. J. Pain* **12**, 900–909 (2008). 18280188 10.1016/j.ejpain.2007.12.015

[52] Hashmi, J. A. & Davis, K. D. Women experience greater heat pain adaptation and habituation than men. *Pain* **145**, 350–357 (2009). 19632779 10.1016/j.pain.2009.07.002

[53] Sullivan, M. J. *et al.* Theoretical perspectives on the relation between catastrophizing and pain. *Clin. J. Pain* **17**, 52–64 (2001). 11289089 10.1097/00002508-200103000-00008

[54] Michaelides, A. & Zis, P. Depression, anxiety and acute pain: links and management challenges. *Postgrad Med.* **131**, 438–444 (2019). 31482756 10.1080/00325481.2019.1663705

[55] Weissman-Fogel, I., Granovsky, Y. & Bar-Shalita, T. Sensory over-responsiveness among healthy subjects is associated with a Pronociceptive state. *Pain Pract.* **18**, 473–486 (2018). 28782305 10.1111/papr.12619

[56] Sava, S. L., de Pasqua, V., de Noordhout, A. M. & Schoenen, J. Visually induced analgesia during face or limb stimulation in healthy and migraine subjects. *J. Pain Res.* **11**, 1821–1828 (2018). 30254484 10.2147/JPR.S160276 PMC6140700

[57] Albu, S., Gómez-Soriano, J., Avila-Martin, G. & Taylor, J. Deficient conditioned pain modulation after spinal cord injury correlates with clinical spontaneous pain measures. *Pain* **156**, 260–272 (2015). 25599447 10.1097/01.j.pain.0000460306.48701.f9

[58] Kumru, H., Soler, D., Vidal, J., Maria, J. & Pascual-leone, A. Evoked potentials and quantitative thermal testing in spinal cord injury patients with chronic neuropathic pain. *Clin. Neurophysiol.* **123**, 598–604 (2012). 21852190 10.1016/j.clinph.2011.07.038

[59] Beese, L. C., Putzer, D., Osada, N., Evers, S. & Marziniak, M. Contact heat evoked potentials and habituation measured interictally in migraineurs. *J. Headache Pain* **16**, 1 (2015). 25564352 10.1186/1129-2377-16-1 PMC5395697

[60] Ruscheweyh, R., Emptmeyer, K., Putzer, D., Kropp, P. & Marziniak, M. Reproducibility of contact heat evoked potentials (CHEPs) over a 6 months interval. *Clin. Neurophysiol.* **124**, 2242–2247 (2013). 23746497 10.1016/j.clinph.2013.05.003

[61] Lütolf, R., Rosner, J., Curt, A. & Hubli, M. Indicators of central sensitization in chronic neuropathic pain after spinal cord injury. *Eur. J. Pain*10.1002/ejp.2028 (2022). 36008094 10.1002/ejp.2028 PMC9826442

[62] Scheuren, P. S. *et al.* Pain-autonomic measures reveal nociceptive sensitization in complex regional pain syndrome. *Eur. J. Pain*10.1002/ejp.2040 (2022). 36130736 10.1002/ejp.2040 PMC10092513

