# Supplementary material for: Understanding inter-individual variability of experimental pain habituation and conditioned pain modulation in healthy individuals
Source: Sci Rep. 2024 Sep 27;14:22070. doi: 10.1038/s41598-024-73158-5 (PMC11436718; doi:10.1038/s41598-024-73158-5)
Supplement: Supplementary file 1 — Supplementary Information. [file 41598_2024_73158_MOESM1_ESM.docx]

## Supplementary information

*
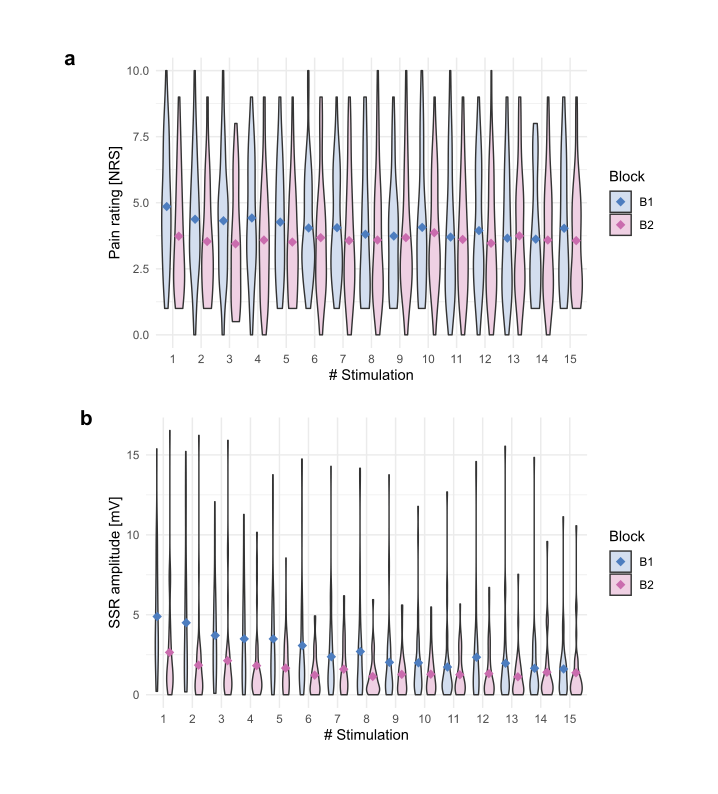
*

Figure S1: Violin plot of single trials over the two stimulation blocks. The x-axis illustrates the number of stimulations for both stimulation blocks (i.e., B1 and B2). The rhombus shows the mean value for each stimulation. (a) Subjective pain rating assessed by a numeric rating scale (NRS) from 0 (no pain) to 10 (maximum pain tolerable). (b) Sympathetic skin response (SSR) amplitude in mV.

Table S1: Detailed statistical results of all correlations corrected for multiple comparisons (N = 21) using the Benjamini-Hochberg correction.

| **Variable 1** | **Variable 2** | **r** | **p** | **p (Benjamini-Hochberg correction)** | **Method** |  |
| --- | --- | --- | --- | --- | --- | --- |
| Pain rating  habituation index | CHEP  habituation index | 0.16 | 0.310 | 0.651 | Pearson |  |
| Pain rating  habituation index | SSR  habituation index | 0.25 | 0.150 | 0.450 | Pearson |  |
| Pain rating  habituation index | Age | -0.27 | 0.075 | 0.315 | Pearson |  |
| Pain rating habituation index | Heat pain threshold (QST) | -0,18 | 0,250 | 0,583 | Pearson |  |
| Pain rating  habituation index | CPM effect  (cold water bath) | 0.50 | 0.001 | 0.025 | Pearson |  |
| Pain rating  habituation index | ‘True’ CPM effect  (corrected) | 0.38 | 0.017 | 0.179 | Pearson |  |
| Pain rating  habituation index | Hospital Anxiety and Depression Scale | -0.18 | 0.240 | 0.583 | Pearson |  |
| Pain rating  habituation index | Pain Catastrophizing Scale | 0.07 | 0.650 | 0.840 | Spearman |  |
| CHEP  habituation index | SSR  habituation index | 0.35 | 0.046 | 0.242 | Pearson |  |
| CHEP  habituation index | Age | 0.01 | 0.960 | 0.970 | Pearson |  |
| CHEP  habituation index | Heat pain threshold (QST) | -0.06 | 0.720 | 0.840 | Pearson |  |
| CHEP  habituation index | CPM effect  (cold water bath) | -0.01 | 0.970 | 0.970 | Pearson |  |
| CHEP  habituation index | ‘True’ CPM effect  (corrected) | -0.07 | 0.660 | 0.840 | Pearson |  |
| CHEP  habituation index | Hospital Anxiety and Depression Scale | -0.06 | 0.720 | 0.840 | Pearson |  |
| CHEP  habituation index | Pain Catastrophizing Scale | 0.03 | 0.870 | 0.962 | Spearman |  |
| SSR  habituation index | Age | 0.27 | 0.100 | 0.350 | Pearson |  |
| SSR  habituation index | Heat pain threshold (QST) | -0.37 | 0.028 | 0.196 | Pearson |  |
| SSR  habituation index | CPM effect  (cold water bath) | 0.16 | 0.380 | 0.725 | Pearson |  |
| SSR  habituation index | ‘True’ CPM effect  (corrected) | 0.12 | 0.510 | 0.824 | Pearson |  |
| SSR  habituation index | Hospital Anxiety and Depression Scale | 0.12 | 0.500 | 0.824 | Pearson |  |
| SSR  habituation index | Pain Catastrophizing Scale | -0.09 | 0.590 | 0.840 | Spearman |  |
| Significant correlations after multiple comparison correction are highlighted in yellow. Abbreviations: CHEP: contact-heat evoked potential; CPM: conditioned pain modulation; SSR: sympathetic skin response; QST: quantitative sensory testing. | | | | | |  |
|  |  |  |  |  |  |  |

Table S2: Sex differences in pain habituation and conditioned pain modulation.

|  | **Female** | **Male** | **t** | **df** | **p** |
| --- | --- | --- | --- | --- | --- |
| Pain rating  habituation index [%] | -9.6 (18.0) | -12.4 (17.6) | 0.52 | 40.8 | 0.607 |
| CHEP  habituation index [%] | -14.1 (19.1) | -18.3 (16.0) | 0.76 | 37.8 | 0.454 |
| SSR  habituation index [%] | -45.8 (28.5) | -42.3 (23.3) | -0.41 | 34.3 | 0.682 |
| Data is presented as mean (SD). Abbreviations: CHEP: contact-heat evoked potential; SSR: sympathetic skin response. | | | | | |
